# Case report: Macrophage activation syndrome in a patient with Kabuki syndrome

**DOI:** 10.3389/fimmu.2024.1412084

**Published:** 2024-07-30

**Authors:** Jingyuan Zhang, Yuanbo Kang, Zenan Xia, Yuming Chong, Xiao Long, Min Shen

**Affiliations:** ^1^ Department of Rare Diseases, Peking Union Medical College Hospital (PUMCH), Chinese Academy of Medical Sciences & Peking Union Medical College; State Key Laboratory of Complex Severe and Rare Diseases, PUMCH; Department of Rheumatology and Clinical Immunology, PUMCH; National Clinical Research Center for Dermatologic and Immunologic Diseases (NCRC-DID), Ministry of Science & Technology; Key Laboratory of Rheumatology and Clinical Immunology, Ministry of Education, Beijing, China; ^2^ Department of Plastic Surgery, Peking Union Medical College Hospital, Chinese Academy of Medical Sciences, Beijing, China

**Keywords:** KMT2D, Kabuki syndrome, macrophage activation syndrome, immunodeficiency, T cells

## Abstract

Macrophage activation syndrome (MAS), is a severe and fatal complication of various pediatric inflammatory disorders. Kabuki syndrome (KS), mainly caused by lysine methyltransferase 2D (*KMT2D*; OMIM 602113) variants, is a rare congenital disorder with multi-organ deficiencies. To date, there have been no reported cases of MAS in patients with KS. This report describes a case of a 22-year-old male with Kabuki syndrome (KS) who developed MAS. This unique case not only deepens the understanding of the involvement of *KMT2D* in immune regulation and disease, but expands the phenotype of the adult patient to better understand the natural history, disease burden, and management of patients with KS complicated with autoimmune disorders.

## 1 Introduction

Macrophage activation syndrome (MAS) is a lethal complication of various inflammatory disorders and is often associated with primary immunodeficiencies (PIDs) (1). Kabuki syndrome (KS) is a rare congenital disorder hallmarked by dysmorphic facial features (including arched eyebrows, long palpebral fissures with eversion of the lower eyelid, and large protuberant or cupped ears), intellectual disability, growth retardation, and other structural and functional defects. Genetic anomalies in lysine methyltransferase 2D (*KMT2D*) and lysine demethylase 6A (*KDM6A*) are implicated in KS. Interestingly, although patients with KS can also exhibit impaired adaptive immunity with significant hypogammaglobulinemia and display autoimmune disorders (2, 3), there are no reported cases of MAS in patients with KS. Here, we present the first report of a KS patient complicated with MAS, indicating a potential association between *de novo* pathogenic variants of *KMT2D* and MAS.

## 2 Case description

A 22-year-old Chinese Han man, clinically diagnosed with common variable immunodeficiency (CVID), was referred to the department of rheumatology and immunology in our hospital due to MAS relapse after medication withdrawal. The patient was susceptible to inspiratory infections during childhood. At the first unprovoked onset of 13, he developed a fever, recurrent respiratory infections, arthritis, and rash. Notably increased ferritin (>2000ng/mL), aspartate aminotransferase (AST) (1930U/L), C-reactive protein (CRP) (66.34mg/L), and erythrocyte sedimentation rate (ESR) (66mm/h) were observed. He was initially diagnosed with systemic juvenile idiopathic arthritis and suspected MAS in the local hospital and treated with methylprednisolone, cyclosporin A, and hydroxychloroquine sulfate. The symptoms improved significantly, with ferritin (288.9ng/mL) and ESR (4mm/h) significantly decreasing. However, pneumocystis pneumonia occurred during steroids, combined with hypogammaglobulinemia in the context, that the patient was suspected of CVID. He began regular intravenous immunoglobulin (IVIG) therapy every three weeks while continuing methylprednisolone, cyclosporine A, and hydroxychloroquine sulfate. He remained stable and was followed up at the local hospital.

At 15, He visited the Department of Pediatrics of our hospital for further evaluation and treatment adjustment. He was considered CVID as consistently low serum IgG ranging from 3.89-4.59g/L. He continued IVIG, methylprednisolone, cyclosporin, and hydroxychloroquine sulfate, and his IgG levels were maintained at 7-8 g/L. During the following 2 years, he remained stable without fever, and regular follow-ups were mainly performed in the local hospital. He gradually discontinued methylprednisolone, cyclosporin, and hydroxychloroquine sulfate in 1.5 years.

**Figure 1:**
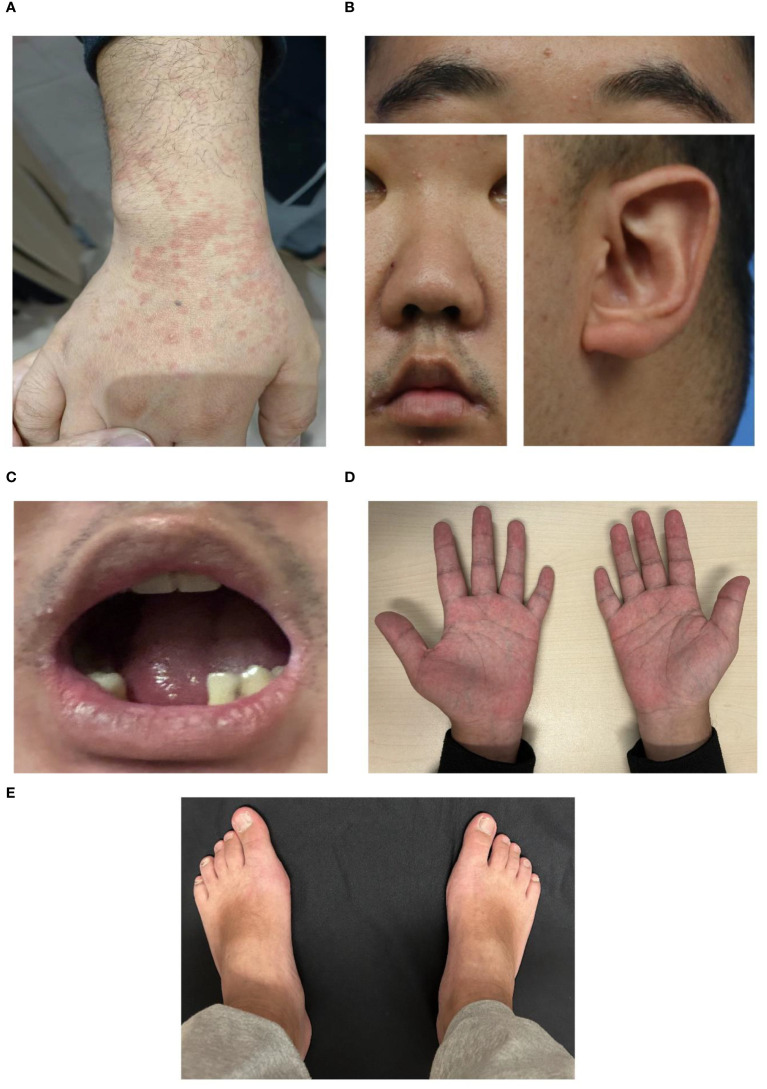
Representative images of the patient with Kabuki syndrome. **(A)** Erythema during a disease flare. **(B)** Representative facial images of the patient. An arched eyebrow, epicanthus, short columella with a slightly narrow nose, thick and narrow lips, and prominent cupped ears were observed in the lateral view. **(C)** Dental anomalies. **(D, E)** brachydactyly. **(D)** Short hands with stubby fingers and dustpan-line finger creases, and short fifth digits were displayed.

Since the age of 17, the patient has been admitted to the local hospital every 2 years for recurrent high fever, erythema of the trunk and limbs (**Figure 1A**), and arthritis, while maintaining regular IVIG every 3-5 weeks as maintenance treatment. Laboratory data showed several elevated ferritin (>1650ng/mL), increased liver enzyme and decreased platelet (PLT) (<100*10^9^/L). Temporary relief was achieved and ferritin returned to normal through pulse therapy of methylprednisolone (500mg/d*3days) and gradual tapering of methylprednisolone plus cyclosporin or tacrolimus over 1-1.5 years, but symptoms recurred six months after withdrawal.

**Figure 2:**
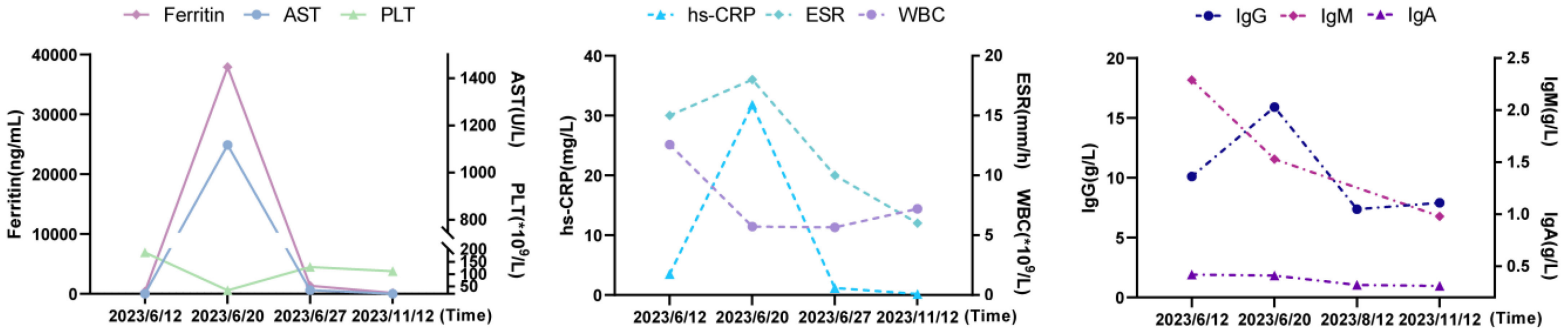
The laboratory data of MAS episode and after-treatment. AST, aspartate aminotransferase; PLT, platelet; hs-CRP, hypersensitive C-reactive protein; ESR, erythrocyte sedimentation rate; WBC, white blood cell; Ig, immunoglobulin.

As recurrent MAS relapse after medication withdrawal, he was transferred to our hospital for further diagnosis at age 22. Laboratory data during this MAS relapse revealed thrombocytopenia (34*10^9^/L, < diagnostic criteria 181*10^9^/L), markedly increased ferritin (37907ng/mL, > diagnostic criteria 684ng/mL), and (AST) (1117U/L, > diagnostic criteria 48U/L). Notably increased Inflammatory indicators ESR (18mm/h, reference value 0-15mm/h) and CRP (31.83mg/L, reference value < 8mg/L) were also observed. Immunologic testing showed decreased serum IgA (0.41g/L, reference value 0.7-4.0g/L), B lymphocytes (21/μL, reference value 180-324/μL), NK cells (77/μL, reference value 175-567/μL), while IgG was normal (15.91g/L, reference value 7-17g/L). The laboratory data of this MAS episode are shown in **Figure 2**. Surprisingly, we found elevated CD8^+^T (1823/μL, reference value 404-754/μL), CD8^+^CD28^+^ (784/μL, reference value 190-392/μL), CD8^+^DR^+^ (1416/μL, reference value 20-178/μL), and CD8^+^CD38^+^ cells (1794/μL, reference value 157-385/μL). An abdomen routine scan showed splenomegaly. Tests for autoimmune indicators and viral infections were normal.

To ascertain the cause of the recurrent fever, trio-whole exome sequencing (WES) was performed and identified a pathogenic “*de novo*” (not present in parents and siblings) missense mutation c.15535C>T, p.Arg5179Cys in the *KMT2D* gene (NM_003482.3, OMIM 602113) (4, 5). Additional questioning revealed that he had hypothyroidism and learning difficulties, with a family history of hypothyroidism but no autoinflammatory diseases, PID, or hemophagocytic lymphohistiocytosis (HLH). Physical examination revealed distinctive features, including arched and broad eyebrows, epicanthus, a shortened and slightly narrow nose, thick and narrow lips, large prominent cupped ears, dental abnormalities, and brachydactyly (**Figures 1B-E**). Facial quantitative analysis, compared to a 20-29-year-old Northern Chinese population, also revealed a classical Kabuki syndrome (KS) facial appearance characterized by a slightly shorter nose, flat nasal bridge, round and blunt nasal tip, and a small, thick mouth (see **Table 1**). The patient was ultimately diagnosed as KS with MAS based on diagnostic criteria (2, 6, 7).

**Table 1:** Facial quantitative analysis results of the patient.

	Patient	20-29 years Northern Chinese Population
Palpebral fissure width	33.4	27.9 ± 1.6
Intercanthal width	34	36.3 ± 3.0
Outer canthal width	100.8	92.0 ± 4.4
Palpebral fissure height	10	10.0 ± 1.0
Midface width	138.3	145.2 ± 4.6
Nasal width	33.1	36.1 ± 3.3
Nasal base width	25.9	25.7 ± 2.8
Nasal height	46.6	53.9 ± 4.4
Nasal length	41.6	48.3 ± 4.7
Nasofrontal angle°	144	132.6 ± 9.1
Nasal tip angle°	102	34.8 ± 5.6
Nasolabial angle°	88	101.0 ± 15.3
Philtrum width	10.5	11.7 ± 1.4
Lip width	36.2	45.4 ± 2.9
Vermilion height	21.8	17.9 ± 2.6
Upper vermilion height	10.8	8.1 ± 1.4
Lower vermilion height	11	9.8 ± 1.6
Upper vermilion area	378	478.4 ± 80.9
Lower vermilion area	349	467.5 ± 74.9

Data presented as mean ± standard deviation.

**Figure 3:**
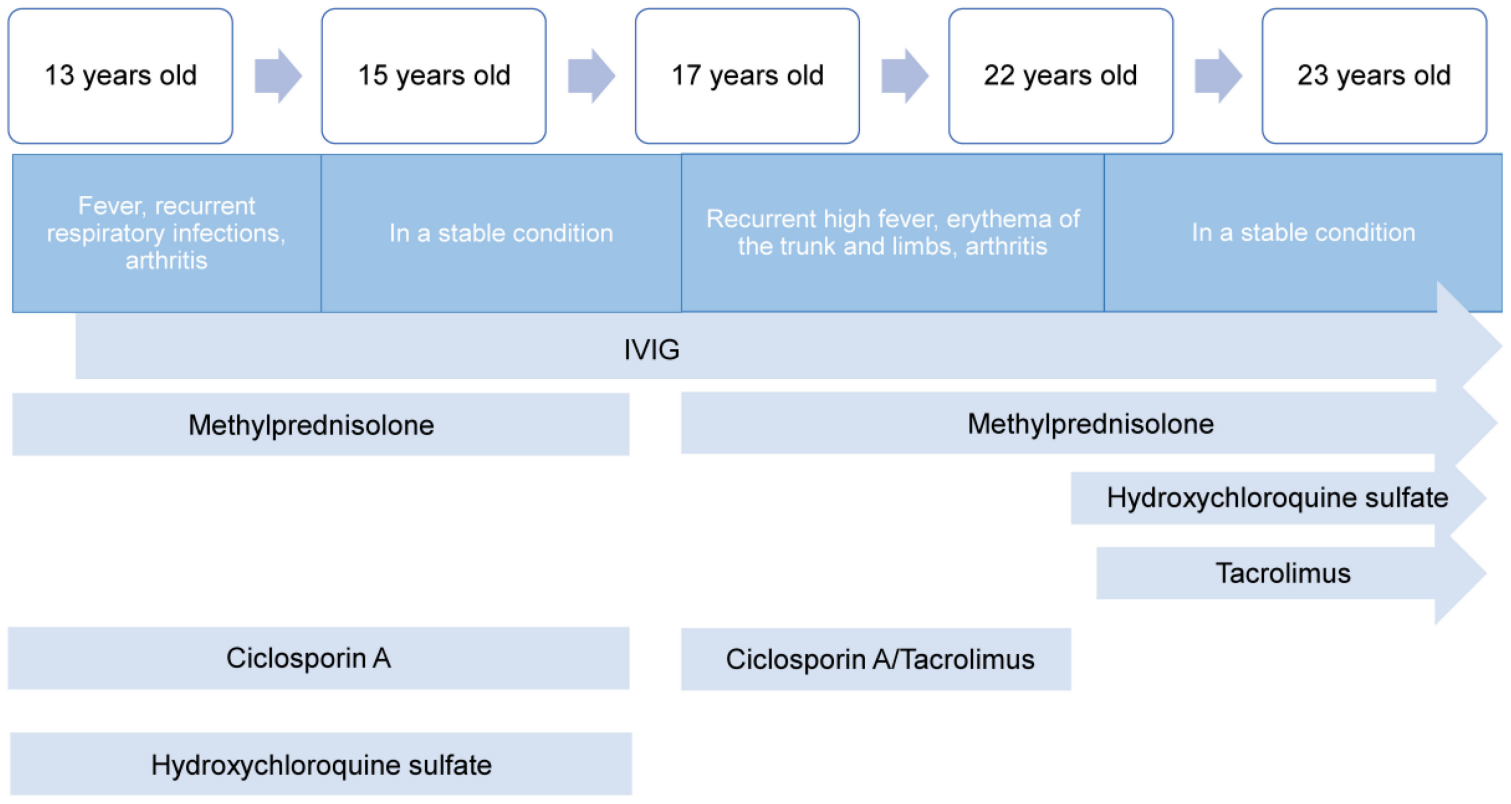
A timeline showing the development of the disease and the treatments received.

He responded well to methylprednisolone pulse therapy (500mg/d*3days), tacrolimus 1mg twice daily, and hydroxychloroquine 1mg twice daily with significant symptoms and inflammation indicators improvement (see laboratory data from 27^th^, June 2023 in **Table 2**). He remained stable without fever while receiving gradual tapered of methylprednisolone, tacrolimus 1mg twice daily, and hydroxychloroquine sulfate 1mg twice daily, alongside regular IVIG with 25-30mg every 3-5weeks, despite low IgG (7.38g/L, reference value 8-18g/L) and IgA (0.31-0.32g/L, reference value 0.7-4.0g/L). Routine blood examination, liver and kidney function, and inflammatory indicators remained normal during the next few months (see laboratory data from 12^th^, August and 12^th^, November 2023 in **Table 2**). **Figure 3** provides a timeline detailing the development of the disease and the treatments received.

**Table 2:** Latest follow-up Laboratory data of the patient.

	2023/6/12	2023/6/20	2023/6/27	2023/8/12	2023/11/12	Reference value
WBC	12.58	5.72	5.65	8.2	7.21	3.5-9.5*10^9^/L
RBC	4.66	5.09	4.25	–	4.67	4-5*10^12^/L
PLT	188	34	129	86	112	100-350*10^9^/L
Ferritin	376.6	37907	1341	–	135	24-336ng/mL
AST	14.2	1117	35	–	20.8	15-40U/L
LDH	356	3271	448	–	–	0-250U/L
hs-CRP	3.5	31.83	1.19	–	0.18	<8mg/L
ESR	15	18	10	–	6	0-15mm/h
IgG	10.1	15.91	–	7.38*	7.9	7-17g/L
IgA	0.42	0.41	–	0.32	0.31	0.7-4g/L
IgM	2.29	1.53	–	–	0.98	0.4-2.3g/L

WBC, white blood cell; RBC, red blood cell; PLT, platelet; AST, aspartate aminotransferase; LDH, lactate dehydrogenase; hs-CRP, hypersensitive C-reactive protein; ESR, erythrocyte sedimentation rate; Ig, immunoglobulin. *reference value 8-18g/L.

## 3 Discussion

Kabuki syndrome is a rare human inborn error of immunity (IEI) involving multi-systems with an estimated frequency ranging from 1:32,000 to 1:86,000 (8, 9) across different ethnicities (10–13). International consensus diagnostic criteria mainly depend on its typical dysmorphic features and pathogenic/likely pathogenic *KMT2D* or *KDM6A* variants. However, facial features of KS, most distinctive between ages 3 and 12, become challenging to identify in infants, adolescents, and adults (2, 14). To address this diagnostic challenge, we utilized facial quantitative analysis to enhance the recognition of facial features in older patients and anticipate future applications of this technology for newborn screening and adjunct diagnosis in adults presenting with symptoms like growth retardation, immune deficiency, and recurrent MAS. Our anthropometric data is limited to the northern Chinese population and may have limited reference value for other populations. Multicenter studies on different populations are warranted to promote the application of this model in patients with KS.

The pathogenesis of MAS is closely linked to persistent Epstein-Barr virus (EBV), and cytomegalovirus (CMV) infection, which trigger abnormal immune stimulation, T-cell activation, and excessive cytokine secretion (1). *KMT2D* plays a key role in terminal B cell differentiation, including somatic hypermutation and class-switch recombination (15, 16). It also epigenetically regulates the gene *ITGB7*, which mediates leukocyte migration to inflamed and non-inflamed regions to provide immune responses (6). *KMT2D* pathogenic variants lead to persistent decreased IgA and IgG levels, NK cells, reduced memory (CD27^+^), and class-switched memory B cells (IgM^−^) with a predisposition to recurrent infections (15, 17, 18). These immunodeficiencies potentially elevate the risk of developing MAS (19). Interestingly, despite normal counts and distributions of T cells and NK cells in most patients with KS (3), our patient study observed an unusual elevation of CD8^+^T, CD8^+^CD28^+^, CD8^+^DR^+^, and CD8^+^CD38^+^. CD8^+^T has been previously reported to be involved in the pathogenesis of MAS (20). Moreover, *KMT2D* is critical for mediating the survival of activation-induced naïve CD8^+^T cells (21), and *KMT2D* variants in colorectal cancer have been linked to increased CD8^+^T cells (22), more immune cell infiltration, and enrichment of immune-related genes and pathways (23). These findings indicated a potential link between *KMT2D* mutations and T-cell dysfunction, leading to MAS, which may be validated in cohort studies using more functional experiments.

KS is also closely associated with various autoimmune disorders (14, 24–26), which may increase in prevalence with age (18). In line with these findings, our patient exhibited significant elevation in anti-thyroid peroxidase antibodies (TPO) antibody, s-thyroid stimulating hormone (TSH), alongside decreased free tetraiodothyronine recently. *KMT2D* encodes for proteins acting in the COMPASS complex, involved in the epigenetic regulation of *FOXP3* (27). *FOXP3* is crucial to the differentiation of naïve CD4^+^ T cells into T-regulatory cells, which contribute to the maintenance of peripheral tolerance (3). Therefore, pathogenic *KMT2D* variants impair the generation, development, and differentiation of regulatory T cells (3), leading to a breakdown of T-cell tolerance, possibly explaining consequent MAS. Further investigation should focus on T cell subsets in a larger cohort of patients with KS to better elucidate the role of *KMT2D* in the regulation of development and pathogenesis of MAS.

Our patients achieved satisfactory disease control with combination therapy of corticosteroid, tacrolimus, and hydroxychloroquine sulfate. Tacrolimus, known for inhibiting T-cell activation in organ transplants (28), supports the role of T-cell activation in KS with concomitant MAS. Early recognition and initiation of potent immunosuppressive therapy can reduce the severity of organ complications. Despite regular intravenous immunoglobulin administration, our patient continued to exhibit low IgA levels, underscoring the need for vigilant monitoring for potential IgA antibody formation (29, 30). Regular testing of immunoglobulin levels is recommended for patients with KS to ensure effective management of the disease.

## 4 Conclusions

This report describes the first case of a patient with KS concurrent with MAS. This unique case provides a clue for the association between *KMT2D* and MAS and the roles of KTM2D in immune regulation and disease. It also expands the phenotype of the adult patient to better understand the natural history, disease burden, and management of patients with KS complicated with autoimmune disorders.

## 5 Patient perspective

The patient and his mother were fully engaged throughout the treatment and told us that his symptoms improved significantly after treatment and remained stable recently. The patient consented to the publication of this case report and written informed consent was obtained.

## Data availability statement

The original contributions presented in the study are included in the article/supplementary material. Further inquiries can be directed to the corresponding authors.

## Ethics statement

The studies involving humans were approved by Peking Union Medical College Hospital. The studies were conducted in accordance with the local legislation and institutional requirements. The participants provided their written informed consent to participate in this study. Written informed consent was obtained from the individual(s) for the publication of any potentially identifiable images or data included in this article. Written informed consent was obtained from the participant/patient(s) for the publication of this case report.

## Author contributions

JYZ: Investigation, Writing – original draft. YBK: Investigation, Writing – original draft. ZNX: Investigation, Writing – review & editing. YMC: Investigation, Writing – review & editing. XL: Supervision, Writing – review & editing, Conceptualization, Investigation. MS: Funding acquisition, Supervision, Writing – review & editing, Conceptualization, Investigation.
